# Electrocochleography in Auditory Neuropathy Related to Mutations in the OTOF or OPA1 Gene

**DOI:** 10.3390/audiolres11040059

**Published:** 2021-11-26

**Authors:** Rosamaria Santarelli, Pietro Scimemi, Chiara La Morgia, Elona Cama, Ignacio del Castillo, Valerio Carelli

**Affiliations:** 1 Department of Neurosciences, University of Padova, Via Belzoni 160, 35121 Padova, Italy; pietro.scimemi@unipd.it (P.S.); elona.cama@unipd.it (E.C.); 2 Audiology Service, Santi Giovanni e Paolo Hospital, Campo Santi Giovanni e Paolo, Castello 6777, 30122 Venezia, Italy; 3 Department of Biomedical and Neuromotor Sciences (DIBINEM), University of Bologna, Via Ugo Foscolo 7, 40123 Bologna, Italy; chiaralamorgia@gmail.com (C.L.M.); valerio.carelli@unibo.it (V.C.); 4 IRCCS Institute of Neurological Sciences of Bologna, Bellaria Hospital, Via Altura 3, 40139 Bologna, Italy; 5 Servicio de Genética, Hospital Universitario Ramón y Cajal, IRYCIS, 28034 Madrid, Spain; idelcastillo.hrc@salud.madrid.org; 6 Centro de Investigación Biomédica en Red de Enfermedades Raras (CIBERER), 28034 Madrid, Spain

**Keywords:** OPA1-related deafness, OTOF-related hearing loss, electrocochleography, cochlear implants, speech perception

## Abstract

Auditory Neuropathy (AN) is characterized by disruption of temporal coding of acoustic signals in auditory nerve fibers resulting in alterations of auditory perceptions. Mutations in several genes have been associated to the most forms of AN. Underlying mechanisms include both pre-synaptic and post-synaptic damage involving inner hair cell (IHC) depolarization, neurotransmitter release, spike initiation in auditory nerve terminals, loss of auditory fibers and impaired conduction. In contrast, outer hair cell (OHC) activities (otoacoustic emissions [OAEs] and cochlear microphonic [CM]) are normal. Disordered synchrony of auditory nerve activity has been suggested as the basis of both the alterations of auditory brainstem responses (ABRs) and reduction of speech perception. We will review how electrocochleography (ECochG) recordings provide detailed information to help objectively define the sites of auditory neural dysfunction and their effect on receptor summating potential (SP) and neural compound action potential (CAP), the latter reflecting disorders of ribbon synapses and auditory nerve fibers.

## 1. Introduction

Auditory neuropathy (AN) is a disorder characterized by alteration of the temporal coding of acoustic signals in auditory fibers with consequent reducction of auditory perceptions [1,2,3]. Disordered synchrony of auditory nerve activity has been suggested as the basis of both the profound alterations of auditory brainstem responses (ABRs) and reduction of speech perception [4].

Auditory neuropathy may be congenital or postlingual in onset. Congenital forms affect the development of language (prelingual AN) [5]. When the onset of AN is delayed to childhood or adult life (postlingual AN), alteration of temporal coding results in severe impairment of speech perception and possible deterioration of acquired language skills [2] Both congenital and postlingual forms of the disorder may be underlain by genetic disorders or result from a huge range of other etiologies (infectious, toxic, metabolic, immune) [2].

Diagnosis relies on decrease of speech perception beyond that expected for the hearing loss, absence or profound abnormality of auditory brainstem responses, and normal outer hair cell activities (otoacoustic emissions and/or cochlear microphonic). However, in some AN patients hearing thresholds are normal and the impairment of speech perception is apparent only in the presence of noise [2,6,7,8]. In these cases, the evaluation of speech perception in noise and psychoacoustical testing (gap detection, frequency discrimination) [3] is mandatory.

Cochlear implantation is the only rehabilitative tool potentially able to restore speech perception in patients affected by pre-synaptic AN [9,10] or post-synaptic AN involving the distal portion of auditory fibers [11,12].

The most well-known forms of AN are due to gene mutations and the mechanisms believed to be involved are functional alterations at pre- and post-synaptic sites, including neurotransmitter release from synapses, spike initiation in auditory nerve fibers and the neural dys-synchrony accompanying demyelination and axonal loss, all resulting in impairment of auditory nerve discharge in response to acoustic stimuli [2,7].

In the last two decades, the identification of many genes involved in the pathogenesis of AN has greatly contributed to the diagnosis and elucidation of the mechanisms underlying this disorder (Table 1). In these disorders AN may present as an isolated hearing disorder (non-syndromic AN) or be associated with multisystem involvement (syndromic AN) [2,7]. In general, most isolated forms of AN are underlain by presynaptic lesions, whereas AN disorders with multisystem involvement are typically due to post-synaptic damage affecting the auditory nerve [7].

We will review how electrocochleography (ECochG) can provide detailed information to help objectively define the sites of auditory neural dysfunction as affecting inner hair cell receptor summating potential (SP) and compound action potential (CAP), the latter reflecting disorders of ribbon synapsis and auditory nerve fibers. This study mostly reflects the experiences from the University of Padua Service of Audiology and Phoniatrics together with the Institute of Neurological Sciences of the University of Bologna and the Servicio de Genética of the Hospital Universitario Ramón y Cajal in Spain.

## 2. Electrocochleography (ECochG)

Electrocochleography (ECochG) recording is acquiring importance in the diagnosis of auditory neuropathy since it allows us to define the details of both neural and receptor responses in the various forms of the disorder [7,35,36]. The identification of specific gene mutations combined with typical electrophysiological patterns may be the key factor in localizing the site of lesions and in revealing how the failure of different molecular processes underlies the varieties of AN.

**Figure 1:**
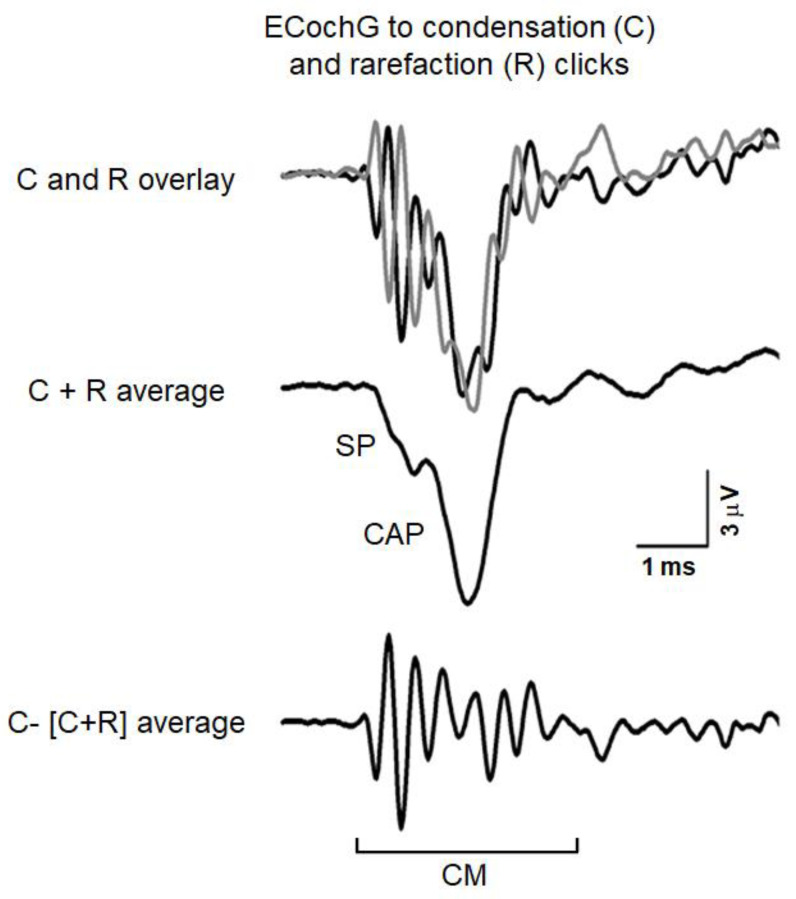
ECochG potentials recorded in a normally-hearing individual at 110 dB nHL. The procedure utilized to separate the cochlear microphonic (CM) from the compound action potential (CAP) and summating potential (SP) is illustrated. The ECochG responses to condensation (C) and rarefaction (R) clicks are superimposed in the top panel. The CAP together with the superimposed SP was obtained by averaging the recordings to condensation and rarefaction clicks (C + R average) through the attenuation of the out-of-phase cochlear microphonics (middle panel). The CM shown in the lower panel results from subtracting the (C + R) average from the ECochG response to condensation clicks. (Reprinted with permission from [35]).

The ECochG potentials evoked in response to acoustic stimuli result from the superimposition of three components, two originating from receptor elements, the cochlear microphonic (CM) and summating potential (SP); and the other, the compound action potential (CAP), arising from auditory nerve fibers ([36] for a review). These components are intermingled in the recordings obtained in response to stimuli of a given polarity and, depending on the type and intensity of acoustic stimulation, cannot easily be distinguished from one another. Figure 1 reports an example of ECochG potentials recorded from the promontory wall in one subject with normal hearing in response to high-level 0.1 ms click stimuli (110 dB peSPL). The recorded signals were amplified (50,000 times), filtered (5–8000 Hz) and digitized (25 µs) and then averaged (500 trials). The procedure of averaging the responses evoked separately by condensation and rarefaction clicks is applied to extract the CAP with the superimposed SP [36]. The top panel displays the ECochG potentials evoked by stimuli of opposite polarity. Superimposed responses to condensation and rarefaction clicks show the phase-reversed CMs intermixed with negative in-phase SP and CAP. Since CM activity is related to the basilar membrane motion, the procedure of averaging the responses evoked separately by condensation, and rarefaction stimuli is applied to extract the CAP together with the superimposed SP. This is shown in the middle panel, where the condensation and rarefaction waveforms have been averaged to cancel the CM and reveal the SP and CAP components. The averaged curve is then subtracted from the response evoked by condensation (or rarefaction) stimuli to obtain the CM (lower panel).

CM is believed to originate mainly from the sum of the extra-cellular components of receptor potentials arising in inner (IHCs) and outer hair cells (OHCs), with the latter contributing more to CM generation because of their greater number [37].

SP is considered to be a gross reflection of the DC component of receptor potentials, which results from asymmetries in the hair-cell transducer function. Because the SP recorded at the round window in chinchillas has been found to decrease in amplitude by over 50% after selective destruction of IHCs, the main contribution to SP generation is believed to arise from activation of IHCs located in the basal cochlear turn Durrant et al. [38]. More recently, Pappa et al. [39] have reported that SP recorded at the round window in both gerbils and human beings results from the contribution of OHC and IHC receptor potentials together with auditory nerve activation. Nevertheless, the SP recorded in normally-hearing individuals in response to click stimuli at high intensity shows peak latencies and mean peak latencies (<1 ms) [36], which rules out synaptic transmission and then neural contribution to SP generation.

CAP results from the weighted sum of the extracellular components of the action potentials generated by individual auditory nerve fibers in response to acoustic stimulation, with the main contribution coming from fibers showing high characteristic frequency and short latency of activation localized to the basal portion of the cochlea [40]. Recently, Bourien et al. [41] have shown that the CAP mostly reflects the contribution of high- and medium-SR (spontaneous rate) fibers, while there is little to no contribution of low-SR fibers.

## 3. Mutations in the OPA1 Gene: A Model of Post-Synaptic AN

Dominant optic atrophy (DOA) is the most common genetic optic neuropathy. The hallmark of the disease is progressive visual loss beginning in childhood [42]. About 60–70% of DOA cases are caused by mutations in the nuclear gene encoding the OPA1 protein, a mitochondria GTPase embedded in the mitochondrial membrane. This protein is involved in fusion of the inner mitochondrial membrane [43], preservation of the structure of mitochondrial cristae [44], maintenance of membrane potential and oxidative phosphorylation [45].

Patients carrying missense mutations localized to the GTPase domain show a syndromic form of DOA associated with several extra-ocular manifestations, such as sensorineural hearing loss, peripheral neuropathy, ataxia, external ophthalmoplegia and myopathy [46,47]. Sensorineural hearing loss affects about 60% of OPA1 patients and AN has been proposed as the mechanism underlying the hearing disorder [11,25,47].

In the last decade, nine hearing-impaired subjects (age range of five–58 years) harboring missense mutations in the *OPA1* gene have been diagnosed and followed up at the University of Padua Service of Audiology and Phoniatrics, while their neurological and ophthalmologic evaluation together with genetic analyses were performed at the Institute of Neurological Sciences of University of Bologna [12]. In all patients vision problems and difficulties in understanding speech began in childhood or adolescence. Audiological assessment showed severe impairment in speech perception, absence or profound abnormalities of auditory brainstem responses and the presence of otoacoustic emissions.

Transtympanic electrocochleography was performed in patients carrying missense mutations in the OPA1 gene at decreasing stimulus intensities from 120 to 60 dB SPL. ECochG waveforms were compared to the corresponding recordings obtained in 20 normally-hearing controls. CM potentials were significantly larger in OPA1 patients compared to controls, thus confirming preservation of outer hair cell activities. We hypothesized that The increase of CM amplitude might result from reduced activity of the efferent system resulting in turn from abnormal activation of auditory fibers.

**Figure 2:**
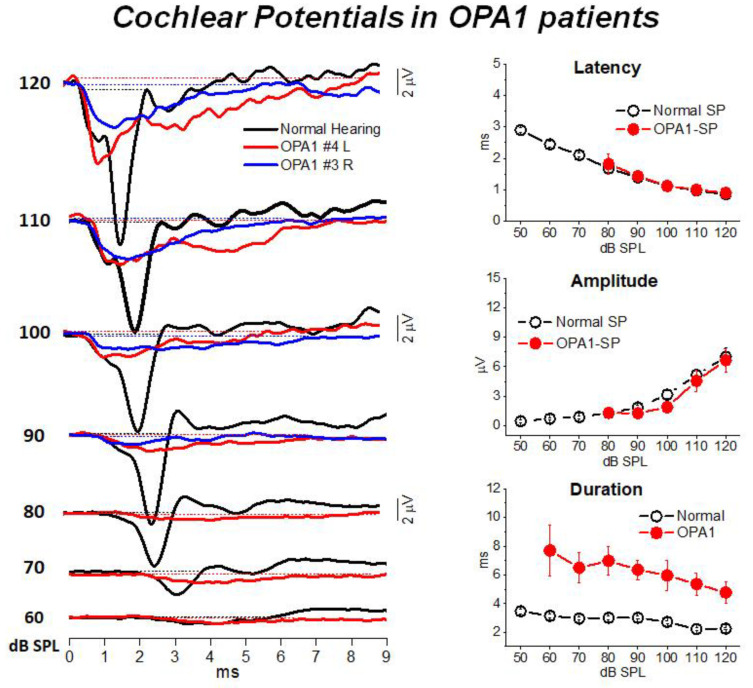
ECochG waveforms recorded from *OPA1* patients. In the left panel the ECochG recordings from two *OPA1*-M patients are superimposed on the corresponding waveforms obtained in one control at decreasing stimulus intensity. In OPA1 patients no CAP was recorded, the ECochG response showing the SP followed by prolonged potential. In this and the subsequent figure time “0” refers to CM onset. In the right panel means and standard errors of peak latency, amplitude and duration of cochlear potentials from OPA1 patients are superimposed on the corresponding values calculated for 20 controls with normal hearing. No differences in SP peak latency and values were found between OPA1 subjects and controls, whereas the duration of cochlear potentials was significantly increased in the group of OPA1 patients. (Modified from [12]).

Figure 2 illustrates the cochlear potentials obtained after CM cancellation from two representative OPA1 patients at decreasing stimulus intensities. The recordings are superimposed to the ECochG waveforms recorded in one normally-hearing control. In the latter, the responses consist of the rapid receptor summating potential (SP) followed by the synchronous neural compound action potential (CAP). Decreasing the stimulus level results in progressive latency increase and amplitude reduction of both SP and CAP peaks. In OPA1 patients the responses recorded at high intensity (120–100 dB SPL) begin with the SP potential, which shows comparable amplitudes and latencies with respect to controls. This finding points to preservation of IHC function. However, differently from controls, no CAP was recorded as SP was followed by a low-amplitude prolonged negative potential, which returned to baseline at 8–9 ms from response onset. This prolonged potential has been interpreted as resulting from abnormal activation of the terminal dendrites of auditory nerve fibers. This hypothesis was confirmed by the reduction in both amplitude and duration of ECochG potentials during high-rate stimulation, which is consistent with their neural generation.

All patients had tried hearing aids without benefit. Eight patients received unilateral cochlear implants in order to improve speech perception by electrical stimulation of auditory fibers through the cochlear implant. Overall, speech perception remarkably improved in all cochlear implant recipients also in the presence of competing noise, although considerable variation of speech perception scores between was observed. Interestingly, no compound action potential was recorded in response to electrical stimulation through the cochlear implant, which confirmed the presence of neural damage. In contrast, brainstem potentials were restored in response to electrical stimulation. This finding supports the hypothesis that the hearing disorder affecting OPA1 patients is underlain by degeneration of the distal portion of auditory nerve fibers. Cochlear implantation restores synchronous activation of auditory pathways by by-passing the site of the lesion.

Overall, these findings indicate that in patients carrying OPA1 missense mutations neural degeneration affecting the terminal dendrites results in a disruption of synchrony of auditory nerve fiber activity. Cochlear implantation improves speech perception in post-synaptic AN associated with OPA1 disease. However, a positive outcome of cochlear implant use was not invariably reported for all forms of post-synaptic AN, variability being possibly dependent on both the sites and extension of the lesion along auditory nerve fibers. Indeed, a variable improvement of speech perception scores has been reported with cochlear implant use in patients with AN associated with Friedreich ataxia [48,49].

The findings collected in families carrying mutations in the *OPA1* gene indicated that combination of findings from genetic research and high-sensitive neurophysiologic recordings plays a crucial role in clarifying both mechanisms and sites underlying the abnormal synchrony of auditory fiber firing.

## 4. Mutations in the *OTOF* Gene: A Model of Synaptopathy

One well-studied form of presynaptic AN is caused by mutations in the *OTOF* gene encoding otoferlin [13,50]. Otoferlin is a transmembrane protein, localized to the synaptic pole in mature IHCs, which has been implicated in multivesicular release at the synapse between the IHCs and auditory nerve fibers [51] as well as in fast vesicle replenishment [52].

Mutations in the *OTOF* gene result in a disrupted function of the ribbon synapses with an impaired multivesicular glutamate release and vesicle replenishment. The majority of subjects carrying mutations in the OTOF gene present with the phenotype of congenital profound hearing loss, the absence of auditory brainstem potentials associated with preserved cochlear hair cell activities (OAEs, CM) [13,50].

**Figure 3:**
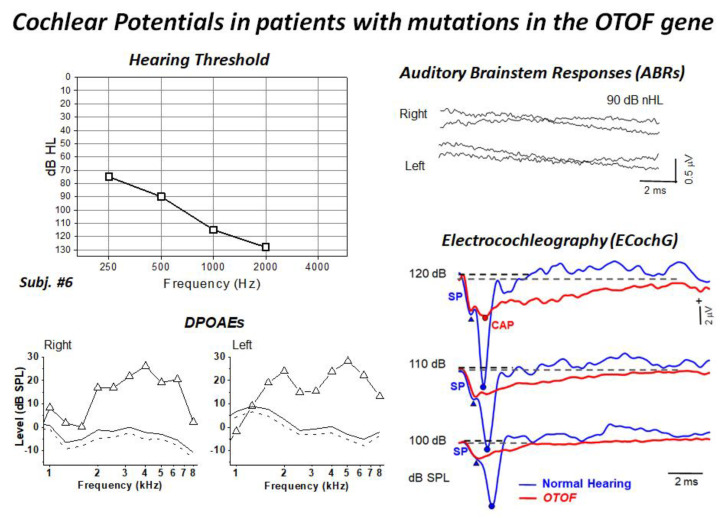
Hearing thresholds and DPOAEs, ABRs and ECochG recordings collected from one child carrying two mutant alleles of the *OTOF* gene. The audiometric assessment performed in the free field using visual reinforcement audiometry indicated profound hearing loss. DPOAEs were recorded from both ears, whereas ABRs were absent. ECochG potentials obtained after CM cancellation are superimposed on the ECochG waveforms obtained in one normally-hearing control. ECochG waveforms begin with the SP, which is followed by a low-amplitude negative potential showing a markedly prolonged duration compared to the control. A small CAP was recorded at high intensity. (Modified from [9]).

In the last ten years, ten hearing-impaired children (age range of four month to three years) harboring biallelic mutations of the OTOF gene have been diagnosed and followed up at the University of Padua Service of Audiology and Phoniatrics, while their genetic analyses were performed at the Hospital Ramón y Cajal in Madrid [9,53]. All children showed profound hearing loss and absent ABRs and presence of distortion product otoacoustic emissions (DPOAEs). Moreover, ECochG recordings showed no differences in CM amplitude between OTOF patients and a group of 20 normally-hearing controls, thus confirming the preservation of OHC function. The audiological and electrophysiological findings collected in a representative patient are displayed in Figure 3. ECochG waveforms obtained after CM cancellation at high intensity (120–100 dB peSPL) are superimposed on the corresponding responses collected from one normally-hearing control. ECochG potentials begin with a rapid negative deflection that peaks at the same SP peak latency as in the normal control, and is of a comparable amplitude, which points to preservation of the IHC function. No CAP was recorded in the hearing-impaired child carrying mutations in the *OTOF* gene, as the SP is followed by a low-amplitude negative potential showing a markedly prolonged duration. Nevertheless, a small CAP is superimposed on the prolonged activity at high intensity in some children.

The prolonged potentials are likely to result from the sum of EPSPs arising in the terminal dendrites, which are dispersed in time as a consequence of the impaired multivesicle release. EPSPs only occasionally reach the threshold for triggering action potentials in some OTOF patients, and are recorded as high-threshold CAPs superimposed on the prolonged activity at high intensity. Therefore, the impaired multivesicular release is likely to result in a lesser probability of synchronized neural spiking and in a reduced signaling to the auditory brainstem pathways in comparison with normal hearing.

All children harboring OTOF mutations diagnosed at our department underwent unilateral cochlear implantation. They showed a remarkable improvement in hearing sensitivity and all reached speech perception scores of 90–100% by one year of cochlear implant use. Moreover, language development was in line with the language skills of their peers using cochlear implants whose deafness was related to causes other than mutations in the *OTOF* gene.

Unlike patients with post-synaptic AN, the implanted children with otoferlin-related deafness showed electrically-evoked auditory nerve potentials (e-CAPs) in response to electrical stimulation through the cochlear implant, consistent with preservation of auditory nerve function. Based on these findings, the outcome of cochlear implantation in patients with presynaptic AN due to OTOF mutations is expected to be successful.

In addition, it is worthy of note that recent studies on animal models of OTOF showed successful preliminary results by using gene therapy [54,55].

## 5. Hearing Dysfunction Related to the OPA8 Locus: A Model of Hidden AN

Hidden auditory neuropathy is characterized by reduced performances in challenging auditory tasks with preservation of hearing thresholds. This condition results from loss of synapses which ensues from degeneration of the unmyelinated portion of auditory nerve fibers [56].

Hidden AN has been observed in ten members of a large Italian family affected by DOA associated with the OPA8 locus (age range 19–72 years) [8,26]. Their neurological and ophthalmologic assessment was performed at the Institute of Neurological Sciences of University of Bologna.

Affected subjects complained of difficulties in understanding speech in the presence of noise. Hearing thresholds and speech perception were normal in the youngest members of this family, whereas the oldest patients showed mild hearing loss at high frequencies and decreased speech perception scores in the presence of noise. OAEs and CM recordings were within normal limits, thus indicating preservation of OHC function in all patients. In contrast, ABRs showed attenuated amplitudes in the youngest patients and severe abnormalities in the oldest. In addition, Wave I amplitude was significantly reduced compared to normally-hearing individuals, consistent with degeneration of IHCs synapses [57].

**Figure 4:**
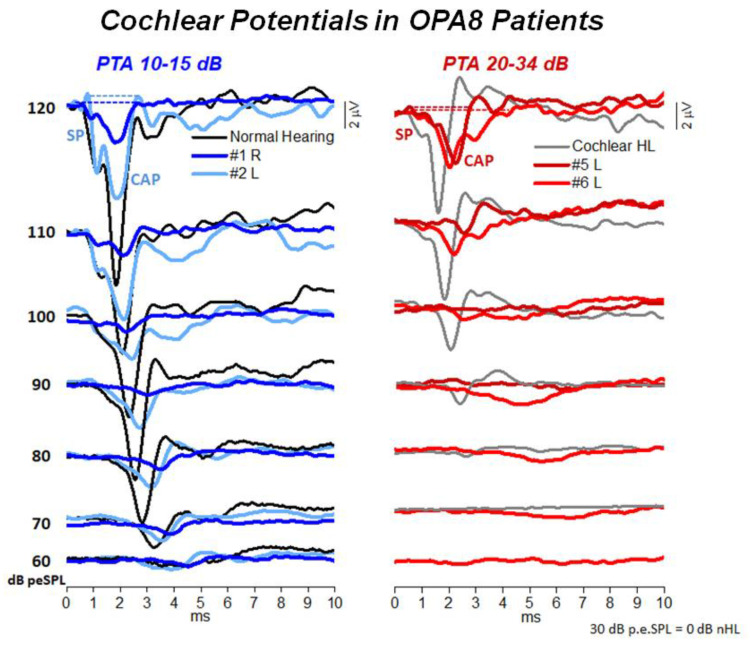
ECochG potentials recorded from patients with OPA8-related DOA. The waveforms obtained from four patients are shown together with the recordings collected in one control at decreasing intensity. The recordings illustrated in the left panel are from two OPA8 patients showing normal hearing thresholds (A), while the ECochG waveforms obtained in two patients showing mild hearing loss are illustrated on the right (B). (Modified from [8]).

**Figure 5:**
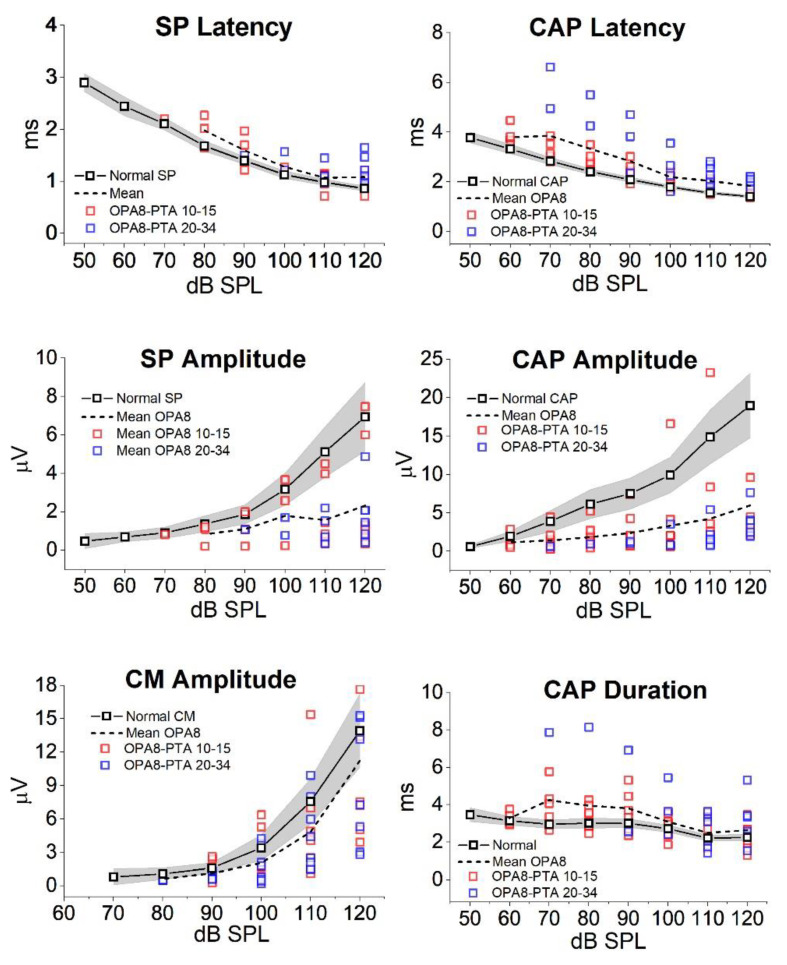
Amplitude, latency and duration of ECochG potentials from OPA8 patients. Individual (colored squares) and mean values (dashed line) of amplitude, latency and duration of ECochG components are reported at various stimulus intensity. Mean values (black open squares), with 95% confidence limits (shadowed areas) obtained in normally hearing controls are superimposed on individual values obtained in OPA8 patients (Modified from [8]).

All subjects were submitted to ECochG recording. Figure 4 reports the ECochG potentials obtained in four OPA8 subjects, two with normal hearing thresholds and two with mild hearing loss, superimposed on the corresponding ECochG waveforms recorded in two controls. Of these, one had normal hearing while the other had a mild hearing loss of cochlear origin. In OPA8 patients the ECochG waveforms begin with the SP potential, which showed reduced amplitudes and increased latencies in comparison to recordings obtained from controls. SP potential was followed by the CAP, which showed a smaller amplitude and an increased peak latency in comparison with controls. The reduction in amplitude and the delay in peak latency of ECochG components were more pronounced in the group of older OPA8 patients showing mild hearing loss (Figure 5). These findings support the hypothesis that in the youngest members of the OPA8 family synaptic damage was far less pronounced [8].

The decrease in amplitude of both SP and CAP points to a decrease of the cochlear output, whereas the increase of both CAP peak latency and duration points to a selective reduction of auditory fibers from the basal portion of the cochlea. This is consistent with the audiometric profile of the oldest OPA8 patients showing mild hearing loss at high frequencies.

These findings indicate that in OPA8 patients hidden AN results from loss of IHCs synapses. At an early stage of the disease, the functional alterations only consist of abnormalities of ABR Wave I while the cochlear potentials show a decrease in amplitude and prolongation in latency of both SP and CAP. At this stage speech perception is relatively preserved. A reduction in speech perception scores and the worsening of cochlear potentials alterations become apparent only with progression of the disease.

## 6. Conclusions and Future Directions

AN disorders associated with gene mutations constitute a group of hearing dysfunctions resulting from different pathophysiological mechanisms. The identification of specific gene mutations combined with typical electrophysiological patterns may be the key factor in revealing how the failure of different molecular processes underlies the varieties of AN. This information is crucial for selecting effective rehabilitative options. In addition, the development of new electrophysiological tools may help in identifying neural and receptor components in the ECochG recordings collected from AN patients [58].

## Figures and Tables

**Table 1:** Genetic disorders underlying AN.

	Locus	Gene	Transmission	Phenotype	Reference
**Isolated AN**					
	2p23–p22	*OTOF*	Recessive	Congenital profound deafness	[13]
	2q31.1–q31.3	*PJVK*	Recessive	Congenital profound deafness	[14]
	13q21–q24	*DIAPH3*	Dominant	Moderate to profound deafness	[15]
	mtDNA	*12S rRNA (T1095C)*		Moderate deafness	[16]
	12q23.1	SLC17A8	Dominant	Progressive	[17]
	3P25.1	TMEM43	Dominant	Post-lingual moderate to profound deafness	[18]
**Non-isolated AN**					
CMT 1A	17p11.2–p12	*PMP22*	Dominant	Mild to severe deafness; demyelinating neuropathy	[19]
CMT 1B	1q22	*MPZ*	Dominant	Mild to severe deafness; demyelinating neuropathy	[20]
CMT 2E	8p21	*NF-L*	Dominant	Normal hearing; axonal neuropathy	[21]
CMT 4D	8q24.3	*NDRG1*	Recessive	Mild to severe deafness; axonal/demyelinating neuropathy	[6,22]
CMT	1p34	*GJB3 (Cx31)*	Dominant	Mild deafness	[23]
CMT 1X	Xp13	*GJB1 (Cx32)*	X-linked Dominant	Demyelinating neuropathy	[24]
ADOA	3q28–q29	*OPA1 (R445H)*	Dominant	Optic neuropathy; moderate deafness	[25]
ADOA	16q21–q22		Dominant	Optic neuropathy, cardiac abnormalities	[26]
AROA	11q14.1–11q22.3	*TMEM126A*	Recessive	Optic neuropathy; mild hearing loss	[27]
Friedreich	9q13	*FXN*	Recessive	Ataxia; axonal neuropathy; optic neuropathy; cardiomyopathy; normal hearing threshold-mild deafness	[28]
AUNX1	Xq23–q27.3		X-linked Recessive	Sensory axonal neuropathy; mild-to-severe deafness	[29]
DDON (Mohr-Tranebjaerg)	Xq22.1	*TIMM8A*	X-linked Recessive	Progressive deafness; dystonia, optic neuropathy; dementia	[30]
LHON (Leber)	mtDNA	*MTND4 (11778mtDNA)*		Optic neuropathy; mild-to-moderate deafness	[31]
Perrault	10q24.31	TWNK	Recessive	Hypogonadism, cerebellar atrophy, cochlear nerve thinning	[32]
USH3A	3q25.1	*CLRN1*	Recessive	Retinitis pigmentosa	[33]
CAPOS	19q13.2	ATP1A3	Dominant	Cerebellar ataxia, areflexia, pes cavus, optic atrophy	[34]
